# Early involvement of peripherally derived monocytes in inflammation in an NMO-like mouse model

**DOI:** 10.1038/s41598-024-51759-4

**Published:** 2024-01-12

**Authors:** Moonhang Kim, Won Seok Kim, Hyeuk Cha, Boram Kim, Young Nam Kwon, Sung Min Kim

**Affiliations:** 1 Biomedical Research Institute, Seoul National University Hospital, Seoul, 03082 Republic of Korea; 2 Department of Neurology, Severance Hospital, Yonsei University College of Medicine, Seoul, 03722 Republic of Korea; 3 Department of Neurology, Seoul National University Hospital, Seoul National University College of Medicine, Seoul, 03080 Republic of Korea

**Keywords:** Demyelinating diseases, Neuroimmunology

## Abstract

Neuromyelitis optica (NMO) is an autoimmune inflammatory disease that primarily affects the optic nerve and spinal cord within the central nervous system (CNS). Acute astrocyte injury caused by autoantibodies against aquaporin 4 (NMO-IgG) is a well-established key factor in the pathogenesis, ultimately leading to neuronal damage and patient disability. In addition to these humoral immune processes, numerous innate immune cells were found in the acute lesions of NMO patients. However, the origin and function of these innate immune cells remain unclear in NMO pathogenesis. Therefore, this study aims to analyze the origin and functions of these innate immune cells in an NMO-like mouse model and evaluate their role in the pathophysiology of NMO. The expression of Tmem119 on Iba1 + cells in brain tissue disappeared immediately after the injection of NMO-IgG + human complement mixture, while the expression of P2ry12 remained well-maintained at 1 day after injection. Based on these observations, it was demonstrated that monocytes infiltrate the brain during the early stages of the pathological process and are closely associated with the inflammatory response through the expression of the proinflammatory cytokine IL-1β. Understanding the variations in the expression patterns of P2ry12, Tmem119, and other markers could be helpful in distinguishing between these cell types and further analyzing their functions. Therefore, this research may contribute to a better understanding of the mechanisms and potential treatments for NMO.

## Introduction

NMO is an autoimmune inflammatory disease that mostly affect optic nerve and spinal cord, and can lead to severe disability of patients through frequent relapse. The pathogenesis of NMO is driven by disease-specific autoantibodies against aquaporin 4 (NMO-IgG)^1,2^. During the acute phase, NMO-IgG infiltrates the CNS perivascular space and binds to AQP4 located on astrocytic endfeet. The destruction of astrocytes (astrocytopathy) through complement-dependent cytotoxicity or antibody-dependent cell-mediated cytotoxicity (CDC/ADCC) triggers an inflammatory response mediated by damage-associated molecular patterns (DAMPs), inflammatory cytokines (such as IL-1β, IL-6, IFN-I), and complement activation (C1q, C3a, C5a)^3–5^. Animal models have been developed to induce inflammatory responses by astrocytopathy, using the passive transfer of patient-derived NMO-IgG and human complement into the brain such as the striatum or ventricles. Additionally, various T cell-based active transfer NMO pathology models have been investigated, including the administration of NMO-IgG alongside experimental autoimmune encephalomyelitis (EAE) or the transfer of encephalogenic AQP4-specific T cells^6,7^. These mechanistic-based models have significantly contributed to understanding the disease pathogenesis, aiding in the development of treatments like complement inhibitors and IL-6 inhibitors^8^. In these animal models, the infiltration of innate immune cells such as monocytes/macrophages and microglia has been identified as a pivotal mechanism contributing to demyelination through myelin debris phagocytosis^9^, becoming a promising therapeutic target.

Monocyte/macrophages and microglia play a crucial role in the acute phase of inflammatory disease of the central nervous system as multiple sclerosis (MS), actively phagocytosing myelin and uniformly distributed throughout the active inflammatory lesion^10,11^. In NMO, activated-demyelinating monocyte/macrophages and microglia characterized by the presence of myelin-laden macrophages are observed along with loss of AQP4 and GFAP^12^. Moreover, activated monocyte/macrophages and microglia secrete chemokines such as MCP-1, MCP-2, and MCP-4, which promote the infiltration of peripheral T cells and leukocytes (monocytes, neutrophils, eosinophils) into the brain^13–16^. They also increase neurotoxicity through the secretion of inflammatory cytokines like IL-1β, TNF-α, IL-6, and the production of C1q via interactions with astrocytes^17,18^. Therefore, it is highly plausible that monocyte/macrophages and microglia play an important role in the early response to injury, contributing to neuroinflammatory demyelination.

Monocytes/macrophages originate from the bone marrow, while microglia derive from yolk sac precursors during CNS development. Despite their distinct origins, classical markers such as Iba1, CD68, F4/80, CD11b, CD45, and Cx3cr1 are commonly shared between monocyte/macrophage and microglia populations, making it challenging to distinguish and analyze their distribution and functions in basic research. Recent advancements in transcriptome analysis and sequencing have facilitated extensive genomic research, leading to the identification of microglia-specific markers such as P2ry12, Tmem119, Siglec H, Olfm3, Fcrls, and Sall1^19,20^. Among these, P2ry12, Tmem119, and Fcrls, which are cell surface markers, have enabled functional analysis of mouse and human microglia using various techniques and methodologies^21^. These markers have been utilized in recent studies to investigate the distribution and functions of monocyte/macrophages and microglia in various neurological disorders, including multiple sclerosis (MS), amyotrophic lateral sclerosis (ALS), ischemia, and Alzheimer's disease^10,22–24^. However, there is a significant lack of research on the distribution and functions of monocyte/macrophages and microglia specific to NMO using these markers. Therefore, in this study, we aim to analyze the distribution and functions of monocyte/macrophages and microglia using the microglia-specific markers P2ry12 and Tmem119 in an NMO-like mouse model.

## Results

### Temporal evolution of immune cell profiles in an NMO-like lesion

**Figure 1:**
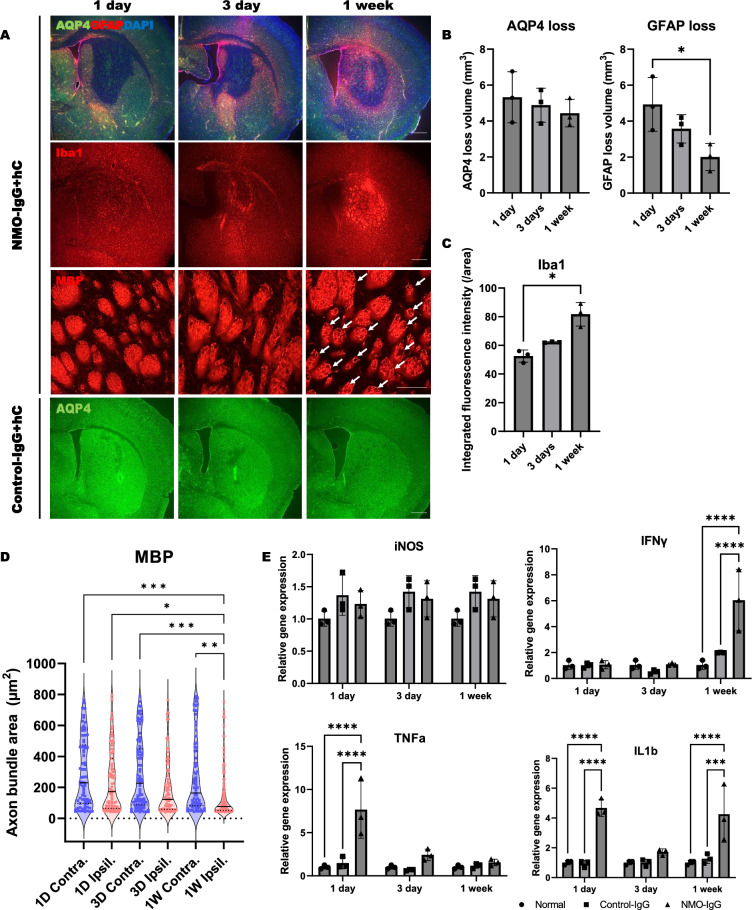
Temporal evolution of astrocytopathy, innate immune cell activation, and myein damage in an NMO-like mouse model. (**A**) Immunostain of injury extent (AQP4 and GFAP), inflammation (Iba1), and MBP loss (MBP) in NMO − IgG + hC and Control − IgG + hC groups at 1, 3, and 7 days. (Scale bar = 500 μm and 100 μm). (**B**) The loss volume of AQP4 and GFAP. (**C**) Quantification of Iba1 + cell activation. (**D**) Measurement of axon buldle size. (**E**) Expression of proinflammatory genes in fresh tissue using qPCR at 1 day, 3 days, and 7 days. (Two-way ANOVA with Tukey's post-hoc test, **p < 0.01, ***p < 0.001, ****p < 0.0001, N = 3).

A pilot experiment was conducted to determine the optimal concentrations of NMO-IgG and human complement. NMO-like mouse model was established using a mixture of 3 μl of 50 mg/ml NMO-IgG and 5% human complement (Supplementary Fig. S1A,B). On 1 day after NMO-IgG + hC injection, staining for AQP4 and GFAP revealed damage to astrocytes (Fig. 1A,B), which was further confirmed by testing astrocyte death using aldh1L1 TG mice (Supplementary Fig. S1C). Activated Iba-1 + monocytes/microglia exhibiting amoeboid morphology were detected at the lesion center 1 day after NMO-IgG injection (Supplementary Fig. S1D). These cells were densely concentrated and proliferating within the axon bundles of the striatum at 1 week (Fig. 1A,C, and supplementary Fig. S1E), coinciding with the gradual collapse of myelin structures (Fig. 1A,D). Upregulation of proinflammatory cytokines were also observed at day 1 (TNFα and IL1β) and also 1 week (IFNγ and IL1β) after injection (Fig. 1E). These results indicated that loss of aquaporin-4 and damage to astrocyte were followed by consecutive activation and/or recruit of innate immune cells with upregulation of proinflammatory cytokines at the lesion center of NMO-like model.

### Monocyte and lymphocyte, rather than microglia, can be actively involved in acute NMO lesion

**Figure 2:**
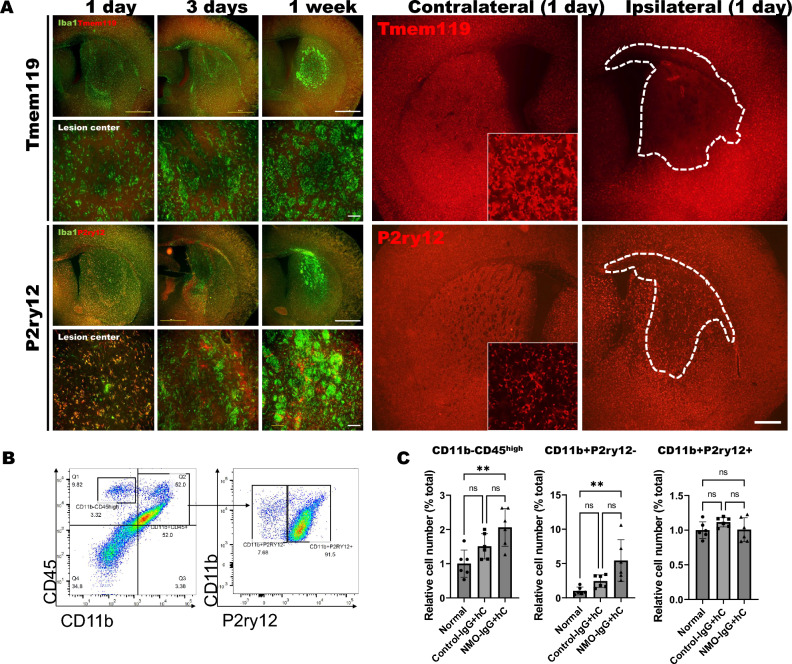
P2ry12 maintains immunoreactivity in early lesion, while Tmem119 does not. (**A**) Immunostaining of Tmem119 (red) and P2ry12 (red) microglia, along with Iba1 (green), at 1, 3, and 7 days post-injury to distinguish infiltrating monocytes/macrophages from microglia and assess their dynamics. Colocalization of each marker was evaluated in the lesion center (Scale bar = 1000 μm and 100 μm). Large images indicate contralateral and ipsilateral expression of Tmem119 and P2ry12 at 1 day after injury. (**B**) Flow cytometry analysis using P2ry12 to differentiate monocytes/macrophages from microglia. CD45 + CD11b + cells were gated, and lymphocytes, monocytes/macrophages and microglia were identified as CD45^high^CD11b−, CD11b + P2ry12− and CD11b + P2ry12 + populations, respectively. (**C**) Each bar graph represents relative number of identified CD11b + P2ry12− monocytes/macrophages, CD11b + P2ry12 + microglia, and CD45^high^CD11b− lymphocytes populations. (Kruskal–Walli's test with Dunn's post-hoc, **p < 0.01 and n.s. = non-significant, n = 6).

We performed immunofluorescence staining of the microglia-specific markers Tmem119 and P2ry12 to differentiate monocytes and microglia and analyze their dynamics (Fig. 2A). Tmem119 expression was not observed at lesion center at 1 day, 3 days, and 1 week after injury. In contrast, P2ry12 staining was observed at 1 day after NMO-IgG injection but disappeared at lesion center after 3 days and 7 days (Fig. 2A). These results indicated that P2ry12 can be more stably expressed at acute inflammatory/activation stage than Tmem119 in NMO pathology, which implies that it can be used as a marker to distinguish monocytes and microglia at 1 day of our NMO-like model.

Next, we attempted to analyze the infiltration/activation of monocytes and microglia into the NMO-like brain lesion using FACS and the microglia-specific marker P2ry12 (Fig. 2B,C) at 1 day after NMO-IgG injection. Only a very small number of CD11b + P2ry12− monocytes/macrophages and CD45^high^CD11b-P2ry12- lymphocytes were observed in the normal brain. The number of CD11b + P2ry12 + microglia remained unchanged, while the infiltration of CD11b + P2ry12− monocytes/macrophages and CD45^high^CD11b − P2ry12− lymphocytes into the inflamed brain was observed (Fig. 2B,C) 1 day after NMO-IgG injection. These results demonstrated that at acute stage of NMO attack, monocyte/macrophage and lymphocyte, rather than microglia, can play as an early player that is involved in inflammation.

### Systemic depletion of monocyte inhibit the expression of the proinflammatory cytokines and loss of myelin in NMO lesion

**Figure 3:**
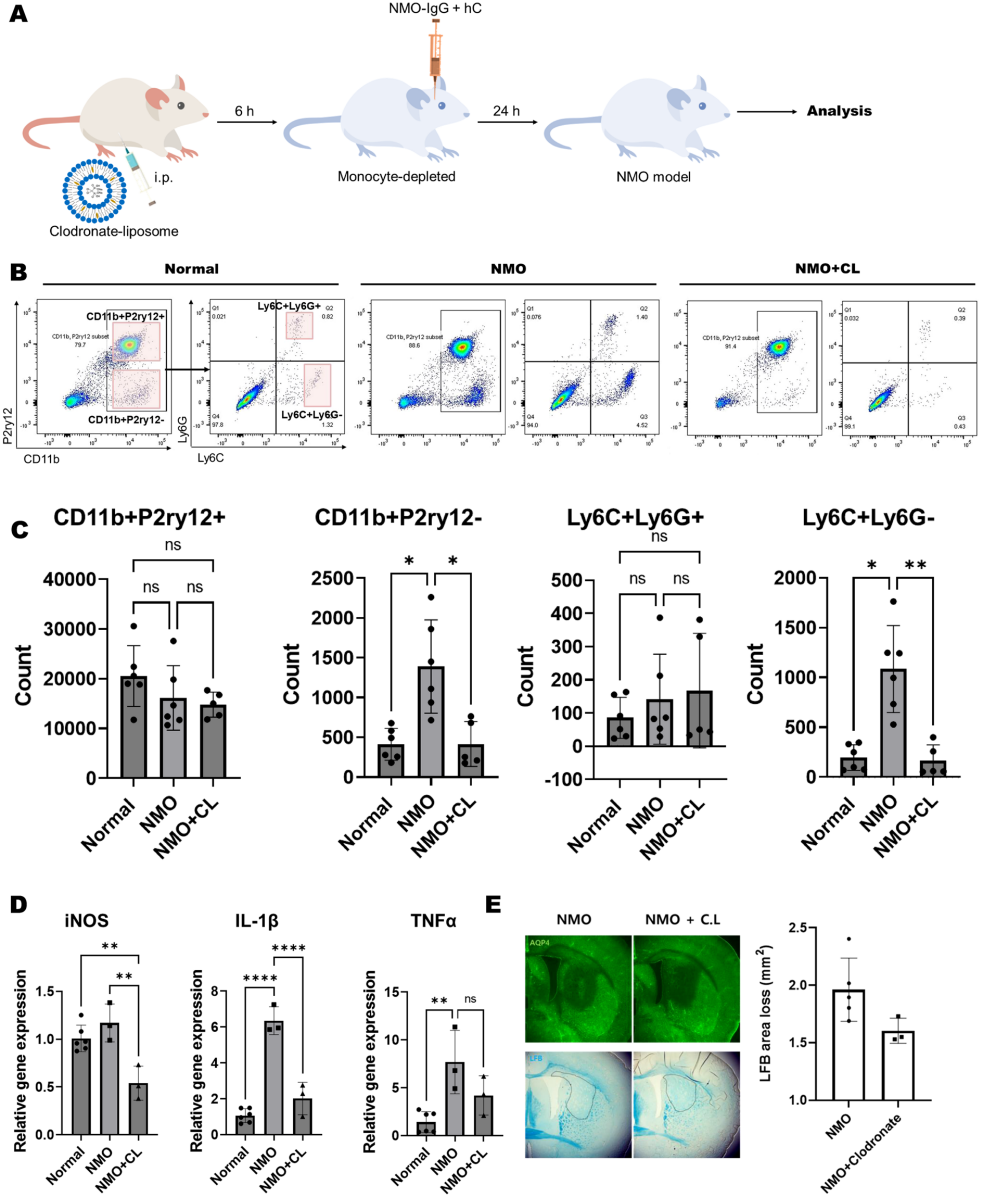
Clodronate depletes monocytes, reduces IL-1β expression and myelin loss. (**A**) Schematic design for monocyte-depleted NMO model. (**B**) Immune cells were isolated from the brains of normal, NMO, and NMO + CL (Clodronate) groups. The dot plot was gated on Live/CD45+, and Ly6C/Ly6G populations were further gated from CD11b + cells. The transparent red squares represent populations of CD11b + P2ry12 + microglia, CD11b + P2ry12− myeloid cells, Ly6C + Ly6G + neutrophils, and Ly6C + Ly6G− monocytes. (**C**) A bar graph displaying differences in cell numbers for each cell type. (Kruskal–Wallis test with Dunn's post-hoc, **p < 0.01, *p < 0.05, and n.s. = non-significant, n = 5–6). (**D**) Relative expression differences of iNOS, IL1β, and TNFα in fresh tissue from each group at day 1 post-injury evaluated by qPCR. (Kruskal–Wallis test with Dunn's post-hoc, **p < 0.01, ****p < 0.0001, and n.s. = non-significant, N = 3). (**E**) Evaluation of demyelination at the injury site through AQP4 immunostaining and Luxol fast blue (LFB) staining, comparing NMO and NMO + CL groups. The dotted line indicates disappeared myelin on LFB staining.

To evaluate the function of infiltrated monocytes in NMO pathogenesis, we systemically depleted monocyte by clodronate treatment in NMO-like mouse model (Fig. 3A). While clodronate treatment did not alter microglia (CD11b + P2ry12+) and neutrophil (CD11b + Ly6c + Ly6G+), it selectively depleted monocyte (CD11b + Ly6C + Ly6G−) population in the CNS of NMO-like model (Fig. 3B,C). Depletion of monocyte in NMO-like model significantly reduced the expression of IL1b and iNOS (Fig. 3D) and also loss of myelin in NMO lesion, whoever it did not affect the loss of AQP4, it significantly inhibit the loss of myelin. (Fig. 3E and Supplementary Fig. S2). These results implied that recruitment of monocyte can be a sequential process that occurs after AQP4 loss by NMO-IgG (or astrocytopathy) and can contribute to the activation of proinflammatory cytokine and demyelination in NMO.

### Monocyte transcriptomic profiles in NMO-like model: infiltrated monocytes express proinflammatory genes in NMO lesion

**Figure 4:**
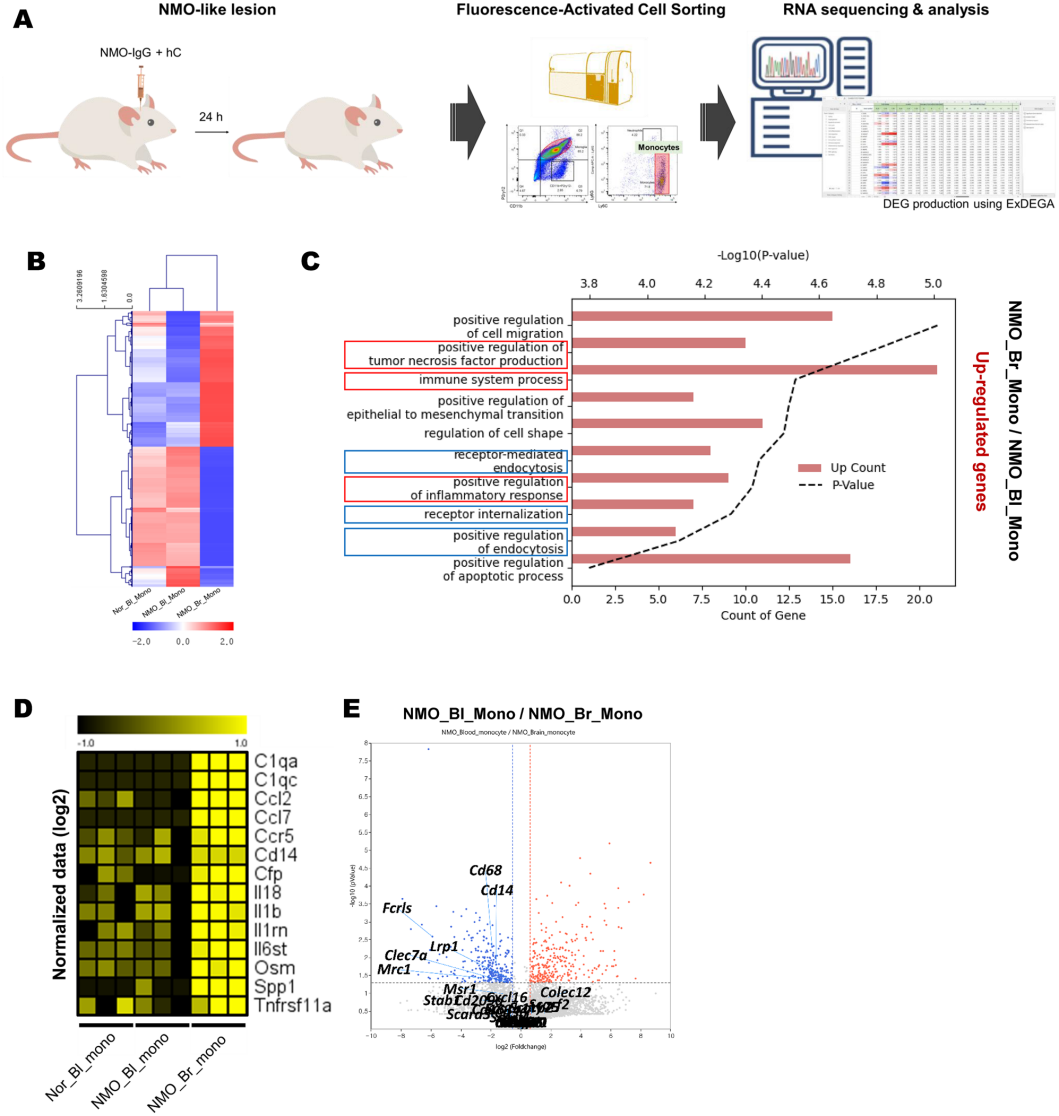
Inflammatory gene and scavenger receptor expression in monocytes infiltrating early NMO lesions increased. (**A**) Schematic design for monocyte sorting and RNA-seq analysis. (**B**) A heat map of RNA-seq data from normal blood monocytes (Nor_Bl_Mono), NMO blood monocytes (NMO_Bl_Mono), and NMO brain monocytes (NMO_Br_Mono) was created using selected genes that showed significant changes (fold change = 1.2, p < 0.05) between NMO_Bl_Mono and NMO_Br_Mono. (**C**) Through the GO analysis of 279 up-regulated genes, the top 10 ontologies were inferred. The red and blue square outlines indicate gene ontologies associated with inflammation and receptor-mediated endocytosis, respectively. (**D**) A heatmap and (**E**) volcano plot represent the expression of inflammation-related genes and scavenger receptors, respectively.

To investigate the functionality of monocytes in NMO pathogenesis, we performed the QuantSeq 3ʹ RNA-sequencing analysis (Fig. 4A and supplementary Fig. S3) and investigated the differentially expressed genes (DEGs) between blood monocytes (Bl_mono) and brain-infiltrated monocytes (Br_mono) in NMO-like model. To obtain monocytes, cells were labeled with CD45, CD11b, P2ry12, Ly6C and Ly6G, and sorted CD45 + CD11b + P2ry12 − Ly6C + Ly6G− cells for monocytes (Fig. 4A). While Nor_Bl_mono and NMO_Bl_mono displayed similar expression patterns, a comparison of DEGs in NMO_Br_mono with NMO_Bl_mono revealed that 279 genes were significantly upregulated in NMO_Br_mono, and 290 genes exhibited significant downregulation (fold change of 1.2, p-value < 0.05; Fig. 4B). The gene ontology (GO) of NMO_Br_mono show upregulate DEG were specifically associated with inflammation (red square outline) and receptor-mediated endocytosis (blue square outline) (Fig. 4C). These results indicate that monocytes infiltrating in NMO-like model are involved in inflammation and acute myelin phagocytosis. Furthermore, various inflammation-related genes showed a significant increase in expression, including *Il1b, Il18, Il6st, Ccl2, Ccl7, Ccr5, Spp1, Osm, Tnfsf11a, CFP, C1qa* and *C1qc* in NMO_Br_mono (Fig. 4D). Similarly, among the 27 scavenger receptors associated with receptor-mediated endocytosis, the expression of *Cd68*, *Cd14*, *Fcrls*, *Lrp1*, *Clec7a*, and *Mrc1* significantly increased in infiltrated monocytes (Fig. 4E). These findings suggest that infiltrating monocytes are associated with the early occurrence of inflammation and demyelination in the NMO-like model.

### Transcriptomic profiles of microglia in NMO-like model and effect of monocytes depletion on it

**Figure 5:**
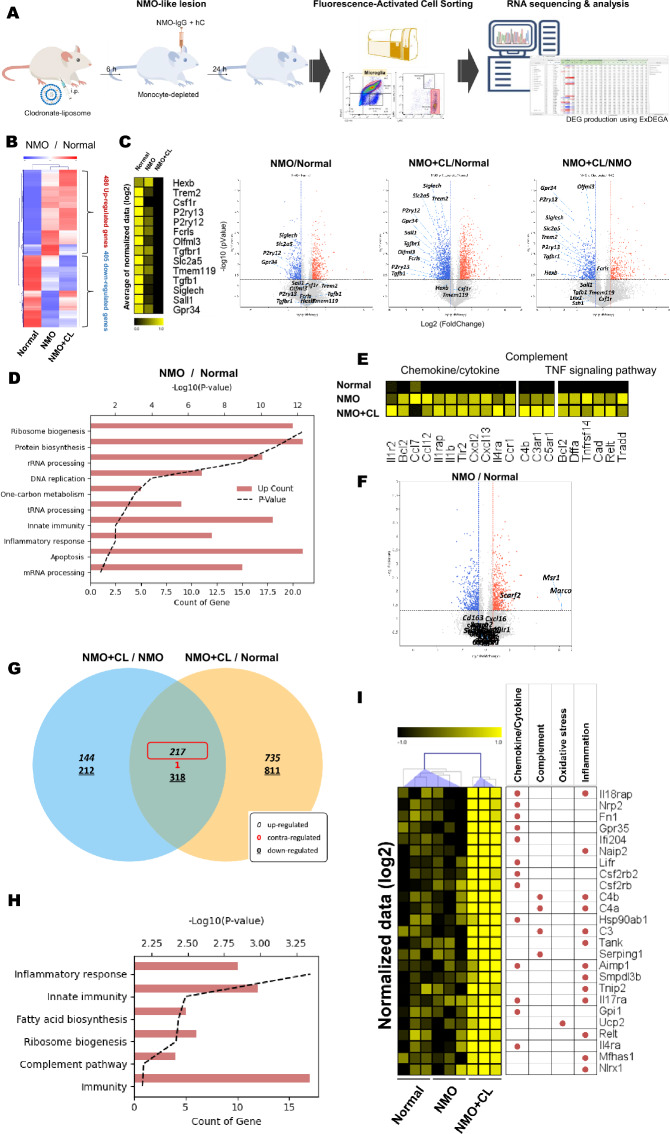
Microglia are activated in early NMO lesions, and monocyte depletion further amplifies this activation. (**A**) Schematic design for microglia sorting and RNA-seq analysis. (**B**–**E**) Depict microglial transcriptome analysis in NMO, while (**F**–**H**) illustrate changes in microglial transcriptome following clodronate treatment. (**B**) A heat map shows DEGs between Normal and NMO (fold change = 1.2, p < 0.05). (**C**) A heat map and a volcano plot related to various microglia-specific marker of DEGs for NMO vs. Normal, NMO + CL vs. Normal and NMO + CL vs. NMO (fold change = 1.2, p < 0.05). Significantly expressed microglial homeostatic markers are highlighted. (**D**) Gene ontology analysis of the upregulated 480 DEGs were inferred. (**E**) The heat map represent the expression of chemokine/cytokine, complement and TNF singling pathway-related genes using average normalized data (log2). (**F**) Volcano plot represent the expression of scarvenger receptors. (**G**) A Venn diagram shows the overlap of upregulated genes in NMO + CL vs. NMO and NMO + CL vs. Normal. (**H**) GO analysis of the 217 overlapping upregulated genes between NMO + CL vs. NMO and NMO + CL vs. Normal. (**I**) A heat map is used to represent the expression of chemokine/cytokine, complement, oxidative stress, and inflammation-related genes within the 217 overlapping upregulated genes.

To analyze microglial function in the NMO-like model, CD45 + CD11b + P2ry12 + cells were sorted, and RNA-seq analysis was performed (Fig. 5A and supplementary Fig. S3A). A total of 885 DEGs were detected, with 480 upregulated and 405 downregulated genes after NMO induction (1.5-fold change, p-value < 0.05; Fig. 5B). Among the downregulated DEGs, functional categorization using DAVID analysis revealed enrichment in "Wnt signaling pathway" annotations (Fig. S4A). Furthermore, microglial homeostasis genes showed decreased expression after the NMO-like model (Fig. 5C). The expression of Wnt-related genes and microglia homeostatic genes decreased in NMO groups compared to the normal group, showing a potential relationship of microglial polarization and activation The GO in the upregulated DEGs were associated with terms related to protein synthesis, DNA replication, and inflammation (Fig. 5D). Genes involved in inflammation, cytokine/chemokine response, complement/receptor, and TNF signaling pathways were upregulated in microglia after NMO-IgG injection, indicating early-stage activation and induction of inflammation through the secretion of various inflammatory cytokines and chemokines (Fig. 5E). An increase in scavenger receptor genes such as *Scarf2*, *Msr1*, and *Marco* was observed (Fig. 5F). To assess the interaction between monocytes and microglia, clodronate was used to deplete monocytes. Clodronate treatment again showed monocyte-specific depletion in our model (Supplementary Fig. S3B). This led to further activation of microglia, as indicated by decreased expression of Wnt genes (Supplemenraty Fig. S4A) and microglial homeostatic markers (Fig. 5C). GO analysis on 217 genes that commonly increased between NMO + CL/NMO and NMO + CL/Normal (1.5-fold change, p-value < 0.05; Fig. 5G,H) showed increased enriched genes associated with inflammatory response, innate immunity, and the complement pathway after clodronate treatment. Additionaly, statistically significant increases in inflammatory genes related to chemokine/cytokine, complement, oxidative stress, and inflammation were observed after clodronate treatment (Fig. 5I). These results indicate that microglia are rapidly activated immediately after astrocytopathy, and they secrete various inflammatory cytokines/chemokines, inducing inflammation. An interesting observation is that microglia become even more activated when monocytes are depleted, which is possibly the result of a regulatory interaction by monocytes. The activation of infiltrated monocytes during the first week of injury seems to be associated with the inhibition of microglial activation and may play a significant role in demyelination.

## Discussion

In this study, we aimed to elucidate the role of blood-derived monocytes in the pathogenesis of NMO. We used NMO-IgG to create NMO-like pathology and observed marker changes in monocytes/macrophages and microglia at different time points. During the acute phase, we confirmed that P2ry12 acted as a specific marker for microglia. Furthermore, we found that monocytes contributed to the exacerbation of NMO pathology through the secretion of inflammatory cytokines, particularly IL1b. In addition, microglia exhibited an inflammatory function regardless of monocyte infiltration, indicating their activation as inflammatory microglia. In this manner, we observed the interplay between monocytes and microglia influencing the disease pathology in the NMO-like animal model. Specifically, we confirmed through transcriptome analysis that monocytes play a role in early inflammation and demyelination.

Monocytes can contribute to the exacerbation of NMO as a secondary damage following astrocytopathy induced by NMO-IgG. Astrocytes release chemokines, and HMGB1 acting as damage-associated molecular patterns (DAMPs), which directly enhance monocyte recruitment^25^ and blood–brain barrier (BBB) leakage^26^. Once infiltrated, activated monocytes promote the production of inflammatory chemokines/cytokines and complement factors, such as Ccl2, Ccl7, Il1b, Il18, C1qa, and C1qc in our study (Fig. 4D). Furthermore, it was also involved in early demyelination through an increase in the expression of scavenger receptors and an increase in receptor-mediated endocytosis (Fig. 4E). This demyelination was reduced after the depletion of pro-inflammatory monocytes by clodronate (Fig. 3E and supplementary Fig. S2). Similar findings were observed in previous studies using chimeric symbiotic mice and a dual reporter system in an EAE mouse model^27^. Ajami et al. reported a positive correlation between infiltrated monocytes and EAE severity, while Ccr2−/− mice showed suppressed EAE progression. Yamasaki et al. demonstrated that monocyte-derived macrophages induced demyelination at EAE onset, and depletion studies showed a moderate delay in disease onset^28^. Taken together, it was evident that monocytes infiltrating in the early pathology of NMO are a key cell type that exacerbates the lesion.

IL-1β plays a role in the expansion of GM-CSF-producing Th17 cells^29^, breakdown of the blood–brain barrier (BBB)^30^, activation of microglia^31^, and promotion of IL-1β production/secretion to amplify inflammation. In our results, *Il1b* showed decreased expression after monocyte depletion (Fig. 3D), whereas microglia showed no change in *Il1b* expression after clodronate treatment (Supplementary Fig. S4B). This implies that monocytes are the primary secretory cells for *Il1b*. IL-1β is known as characteristic biomarkers in the serum and cerebrospinal fluid (CSF) of NMO patients^32,33^. Additionally, IL-1β increased in the serum of patients with acute stages of NMO^34^. These findings support our study's assertion that monocytes induced IL-1β play an important role in the early stages of NMO. The current treatment for NMO primarily focuses on long-term management and prevention of relapses. However, targeting monocytes for acute management in patients with relapses is predicted to offer therapeutic potential that could reduce the severity. Additionally, IL-1β is known not only as a major cytokine amplifying inflammation but also as a regulator of proliferation and differentiation in Oligodendrocyte Progenitor Cells (OPCs)^35,36^. In this study, delayed expression of *Il1b* was observed at 1 week, but unfortunately, the cellular source of this expression remained unidentified. In-depth research into this delayed expression could significantly contribute to understanding the regulation of demyelination and remyelination in demyelinating diseases.

This study confirmed the substantial involvement of monocytes in inflammation and demyelination in NMO early lesions. Simultaneously, a regulatory function decreasing microglial activation was also observed after clodronate treatment (Fig. 5G,H). The analysis of selected signature genes related to pro-inflammatory (*Il6*, *Tnf*) or anti-inflammatory (*Arg1*, *Tgfb1*)^37^ responses revealed increased expression of *Arg1* and *Tgfb1* after infiltration, while *Il6* and *Tnf* decreased in the brain following infiltration (Supplementary Fig. S4C). Further detailed research is needed to determine whether monocytes perform both of these functions or if there is a distinct population contributing to these effects. One hypothesis is that the P2ry12 − CD11b + Ly6G − Ly6C + cell population sorted for this study might encompass myeloid-derived suppressor cells, which could potentially explain the observed phenomenon. Despite enhanced microglial activation after clodronate treatment, the importance of monocytes in contributing to acute lesion development remains unchanged, as evidenced by the reduction in myelin damage. Additionally, the significance of the increase in *Il1b*, irrespective of the decrease in *Il6* and *Tnf*, remains unchanged.

Microglia, like monocytes, are known to play a crucial role in the pathogenesis of NMO^38^. The exact mechanisms and stimuli leading to microglia activation are not fully understood, but our study showing that monocyte depletion does not reduce microglia activation, suggests that microglia, similar to monocytes, may act as the first responders to astrocytic chemokines/cytokines and DAMPs. Moreover, recent research has reported that C3a, produced by astrocytes in precytotic tissue injury in NMO, can activate microglia^18^. Our microglia transcriptome analysis supports this microglia-astrocyte interaction, as it showed an increase in C3aR expression (Fig. 5E). Additionally, the immediate reduction in Wnt signaling and homeostatic microglia markers after NMO-IgG injection well indicates acute microglia activation (Fig. 5C, Supplementary Fig. S4A). Microglia are known to be involved in various functions in NMO, including inducing neuronal damage, clearing debris, and promoting inflammation. In our study, microglia exhibited an increased enrichment of cell proliferation, protein synthesis, and inflammation-related genes after NMO-IgG injection (Fig. 5D). Moreover, the upregulation of inflammatory chemokines/cytokines and complement expression well strongly indicates their role as inflammation-associated microglia (Fig. 5E). However, due to the results showing the disappearing pattern of the P2ry12 marker, we were unable to determine microglia's roles in the chronic phase.

Among homeostatic microglia markers, P2ry12 and Tmem119, as cell membrane proteins, have the advantage of enabling analysis through immune reactivity techniques such as FACS, immunostaining, and Western blotting. Tmem119 has recently been identified as a microglia-specific marker capable of distinguishing macrophages from microglia in both human and murine tissues^21,22^. However, the discrepancies between *Tmem119* mRNA and protein expression, as well as its disappearance in CNS disease animal models with tissue damage, raise concerns about its reliability as a marker^39^. On the other hand, P2ry12 exhibited a different expression pattern, maintaining immunoreactivity on day 1 after injection and disappearing on days 3 and 7 (Fig. 2A). This pattern is consistent with observations in post-mortem brain tissue of MS patients, where P2ry12 remains in chronic-active lesions while both P2ry12 and Tmem119 are lost in active lesions^24^. These differing expression patterns suggest that P2ry12 is less affected by lesion activity and may serve as a more reliable marker for microglia during the acute phase.

In most studies that utilized P2ry12 and Tmem119 to distinguish and analyze monocytes/macrophages from microglia, immunoreactivity in active lesions disappeared, similar to our study. However, by considering the expression characteristics based on lesion activity, as demonstrated in our research, it is possible to effectively distinguish between monocytes/macrophages and microglia. Therefore, a primary focus should be on understanding the lesion activity of the disease under investigation, and the timing of analysis also becomes a critical factor. Additionally, a comprehensive understanding of P2ry12 and Tmem119 in microglia should be accompanied by in-depth research, and the exploration of new markers that can distinguish monocytes/macrophages from inflammatory microglia is also crucial.

Finally, this study demonstrated that monocytes infiltrating during the acute stage can play a critical role in initiating early inflammation. Additionally, we showed an increase in the secretion of Il1b from infiltrated monocytes. The upregulation of Il1b during the acute attack is a common feature in most demyelinating diseases. Further additional research is needed to determine whether therapies targeting monocyte/macrophage and Il1b in NMO patients can effectively prevent relapses and reduce disability in the clinical field.

## Methods

### Mice

All experiments were performed using female mice. We established an NMO-like animal model using female wild-type C57BL/6N mice (purchased from Orient Bio, Seongnam, Korea) at 12 weeks of age.The presence or absence of astrocyte damage was confirmed using Aldh1l1–EGFP–Rpl10a transgenic mice [B6; FVB-Tg (Aldh1l1/EGFP/Rpl10a) JD130Htz/J] (#030247, the Jackson Laboratory). All animal experiments were performed in compliance with the standards operating guidelines and approved by the Institutional Animal Care and Use Committee at Seoul National University (SNU-220215-1). All the animal experiments in this study are conducted in accordance with ARRIVE (Animal Research: Reporting of in vivo) guidelines.

### Patient-derived NMO-IgG preparations

Purified NMO-IgG and Control-IgG were prepared following the methods described in a previous study^40^. Briefly, IgG purification was performed using a commercially available kit (Amicon® Pro Affinity Concentration Kit–IgG, Billerica, MA) in accordance with the manufacturer's protocols. Plasma protein was added to a protein A resin-embedded exchange device and incubated at room temperature for 60 min with gentle agitation. Following the protein-binding step, the resin was centrifuged at 1000×*g* for 1 min. For the wash step, 1.5 ml of Bind/Wash Buffer was added, and the mixture was centrifuged again at 1000×*g* for 1 min. Elution buffer was added to the samples, and the mixture was centrifuged at 1000×*g* for 2 min. The pH was adjusted by adding neutralization buffer. The concentration of NMO-IgG was measured using the NanoDrop One, and aliquots of 100 mg/ml NMO-IgG were prepared in 35 μl volumes and stored until use. The present study was approved by the SNUH Institutional Review Board (approval number: H-1902-083-1010). All patients provided written informed consent before participating. All methods were performed in accordance with the relevant guidelines and regulations.

### NMO-like mouse model

A NMO-like mouse model was created by unilateral intrastriatal injection of NMO-IgG and human complement (S1764, Sigma). The mice were anaesthetized using Zoletil (20 mg/kg, Zolazepam + Tiletamine) and Xylazine (10 mg/kg) and mounted onto a stereotaxic Instrument (#900, Kopf). A Midline incision was made to expose bregma, and a burr hole was created on the right side at the 0.5 mm of anterior, 2 mm of lateral from bregma. 10 μl gas-tight glass Hamilton syringe with a 33 g needle (#80008, Hamilton) was inserted 2.5 mm deep to infuse NMO-IgG and complement mixture. To determine optimal concentration of mixture, we tested 0, 10, 30 and 50 mg/ml NMO-IgG along with 0, 1, 5 and 10% complement. The optimal mixture, consisting of 3ul of 50 mg/ml of NMO-IgG with 5% complement, was injected at a rate of 1ul/min. Following the injection, a resting period of 2 min was provided to ensure sufficient diffusion of the injected material. The scalp was then closed with 6-0 silk suture. We established experimental groups comprising non-treated normal mice, as well as groups receiving injections of Control − IgG + hC and NMO − IgG + hC mixtures at equivalent concentrations into the unilateral striatum. These groups were sacrificed at 1, 3, and 7 days post-injection for immunofluorescence, qPCR, and flow cytometry analyses. For the monocyte/macrophages depletion experiment, groups were categorized as Normal, NMO, and NMO + CL. These groups were sacrificed at 1 day post-injection for flow cytometry, qPCR, immunofluorescence, and western blotting analyses. Additionally, at 1 week post-injection, LFB (luxol fast blue) staining and western blotting were conducted to assess the extent of demyelination. For the RNAseq of monocyte experiment, blood monocytes from Normal and NMO mice, as well as brain monocytes from NMO mice, were sorted from the immune cells and their transcriptomes were compared at 1 day post-injection. The RNAseq study of microglia included Normal, NMO, and NMO + CL groups, where brain immune cells were isolated for comparative transcriptome analysis. Detailed experiments and sample number in each group are provided in Supplementary Table S1.

### Tissue preparation and immunofluoresence

At 1 day, 3 days and 7 days after injury, mice were anesthetized using Zoletil + Xylazine and perfused fixed through the left cardiac injection of PBS followed by 4% paraformaldehyde (PFA, PC2031-100, Biosesang, Korea). Brain tissue was harvested and post-fixed in 4% PFA. The tissues were then dehydrated using a progressive treatment with 10–30% sucrose, embedded in frozen section compound (3801480, Leica, Richmond, IL, USA), and frozen at − 80 ℃. Serial floating sections, with a thickness of 30 μm, were preserved in a storage solution (30% Glycerol in PBS) in 24-well plates at − 20 ℃ until use.

Sections were immunostained with the following primary antibodies: goat anti-AQP4 (1:500, sc-9888, Santa Cruz), rabbit anti-AQP4 (1:500, AQP-014, Alomone), rabbit anti-GFAP (1:500, ab7260, Abcam), rat anti-MBP (1:500, ab7349, Abcam), goat anti-Iba1 (1:200, NB100-1028, Novus), rat anti-P2ry12 (1:200, 848002, Biolegend), and rabbit anti-Tmem119 (1:100, ab209064, Abcam). The sections were then incubated with the appropriate secondary antibodies for each species: FITC-conjugated donkey anti-goat IgG (H + L) (1:200, 705-056-147, Jackson ImmunoResearch), Alexa Fluor® 594-conjugated donkey anti-rat IgG (H + L) (1:200, 712-585-153, Jackson ImmunoResearch), and Alexa Fluor® 594-conjugated donkey anti-rabbit IgG (H + L) (1:200, 711-586-152, Jackson ImmunoResearch) The sections were coverslipped with VECTASHIELD® Antifade Mounting Medium (H-1200, Vector Laboratories) and immunofluorescence was visualized using a Nikon ECLPSE N*i*-E microscope. The loss volumes of AQP4 and GFAP were measured through serial sections at 290 μm intervals. The fluorescence intensity of Iba1 immunoreactivity was measured using ImageJ. The extent of axonal bundle damage in the MBP-stained sections was analyzed using the Analyze Particles plug-in in ImageJ (Supplementary Fig. S1F).

### Quantitative PCR

Fresh tissue samples were collected from the Normal group (n = 3), Control − IgG + hC group (n = 3), and NMO − IgG + hC group (n = 3) at 1 day, 3 days, and 7 days post-injury, respectively. The samples were obtained from the 2 mm thickness of the ipsilateral hemisphere around bregma using a 2 mm biopsy punch to isolate the striatum tissue. Subsequently, these collected samples were stored at − 80 °C until further processing. Total RNA was isolated using the RNeasy Mini Kit (74104, Qiagen), and cDNA synthesis was performed using the AccuPower® CycleScript™ RT PreMix & Master Mix (74004, Bioneer). The following primers were used: forward 5ʹ-CTTCCCAGGATGAGGACATGAGCAC-3ʹ and reverse 5ʹ-TCATCATCCCATGAGTCACAGAGG-3ʹ for *IL1B*; forward 5ʹ-AGCCGATGGGTTGTACCTTG-3ʹ and reverse 5ʹ-GTGGGTGAGGAGCACGTAGTC-3ʹ for *TNF*; forward 5ʹ-CCCTTCAATGGTTGGTACATGG-3ʹ and reverse 5ʹ-ACATTGATCTCCGTGACAGCC-3ʹ for *NOS2*; forward 5ʹ-ATGAACGCTACACACTGCATC-3ʹ and reverse 5ʹ-CCATCCTTTTGCCAGTTCCTC-3ʹ for *IFNG*. Quantitative real time PCR analyses were performed using Real-Time PCR instrument system (ABI7500, apppliedBiosystems) with PowerUP™ SYBR™ Green master mix (A25741, apppliedBiosystems) The expression of genes were normalized to that of housekeeping gene GAPDH and quantified using the 2^−∆∆Ct^ method.

### Flow cytometry analysis of CNS immune cells

The mice were anesthetized and then transcardially perfused with cold PBS. The brains were isolated and placed in 2 ml of ice-cold Hank's Balanced Salt Solution (HBSS). To facilitate dissociation, 123 μl of Liberase TL (05401020001, Roche) and 5 μl of 100 mg/ml DNase I (DN25, Sigma) were added to the brain tissues and incubated for 30 min at 37 ℃. After enzymatic dissociation, the cells were resuspended in 30% Percoll (17089101, Citiva, Sweden) and centrifuged for 25 min at 2850 rpm without a brake. Myelin was removed, and the pelleted cells were washed with HBSS. Red blood cells (RBC) were lysed using RBC lysis buffer (420301, BioLegend), and the cells were then counted using AO/PI (CS2-0106, Nexelom Bioscience, MA, USA) staining and a Fluorescent cell counter (Cellometer K2, Nexelom Bioscience, MA, USA).

CNS immune cells were stained with the LIVE/DEAD™ Fixable Blue Dead Cell Stain Kit (L34962, Invitrogen) and incubated with Fc-Receptors blocker (101302, BioLegend). The cells were then labeled with the following antibodies: PECy7 anti-CD45 (561868, BD), FITC anti-CD11b (101206, BioLegend), PE anti-P2ry12 (848004, BioLegend).

### Monocyte/macrophage depletion

Mice underwent monocyte depletion through intraperitonial injection of 200ul Clophosome^®^-A-Clodronate Liposomes (F70101C-A, 7 mg/ml, FormuMax) 6 h prior to NMO − IgG + complement injection. Mice were divided into three groups: Normal, NMO (NMO − IgG + complement), and NMO + CL (NMO − IgG + complement + clodronate liposome). After inducing NMO damage, the mice were sacrificed at 1 day post-injury to collect brain tissues. The obtained brain tissues were subjected to FACS analysis using FITC anti-CD11b (101206, BioLegend), PE anti-P2ry12 (848004, BioLegend), APC anti-Ly6G (127614, BioLegend), BV711 anti-Ly6C (128037, BioLegend) antibodies and Zombie Violet™ (423113, BioLegend) for Live/Dead cells. Additionally, the gene expression of *IL1B*, *TNF*, and *IFN* were analyzed using quantitative PCR.

LFB staining was performed using the VitroView™ Luxol Fast Blue Stain Kit (VB-3006, VitroVivo Biotech, USA) according to the manufacturer's instructions. Briefly, the sections were immersed in a defat solution overnight. Then, the sections were hydrated in 95% ethanol. Subsequently, the sections were stained with a Luxol Fast Blue solution and differentiated in a lithium carbonate solution. Finally, the sections were washed with distilled water to remove any remaining stains.

The samples, obtained using a 2 mm biopsy punch from the 2 mm thickness of the ipsilateral hemisphere around bregma to isolate striatum tissue, were stored at − 80 °C until further processing. For MBP expression analysis at 1 day and 7 days, brain tissues were homogenized and extracted in RIPA buffer (RC2002-050-00, Biosesang) containing a protease inhibitor (11836170001, Roche).The protein concentration was quantified using the Pierce™ BCA Protein Assay Kit (23227, Thermo Inc.). Subsequently, protein denaturation was achieved with 4 × Laemmli sample buffer (#1610747, Bio-Rad), and 5 μg of protein was loaded per well. The proteins were separated using a 12% polyacrylamide gel and transferred to a PVDF membrane using the iBlot2 system (Invitogen). After blocking with 5% skim milk, the membrane was probed with rat anti-MBP antibody (1:1000, ab7349, Abcam) and mouse anti-actin antibody (643802, BioLegend). MBP was detected using HRP-conjugated goat anti-mouse IgG antibody (1:10,000, 405306, BioLegend) and HRP-conjugated goat anti-rat IgG antibody (1:10,000, ab205720, Abcam), followed by visualization using a chemi image system (Amersham 680, GE Lifesciences).

### RNA-seq analysis of sorted monocyte and microglia

The immune cells from the brain tissue of the Normal and NMO groups on day 1 were isolated using the method described above. Each experimental group consisted of three independent biological replicates derived from a pool of five samples. Mouse peripheral blood mononuclear cells (PBMC) were isolated using density gradient medium Lymphoprep™ (#07861, stemcell technologies, Germany). For purification of monocytes, CD45 + P2ry12 − CD11b + Ly6G − Ly6C^high^ Cells were sorted using BD FACsymphony™ S6 cell sorter. For microglia sorting, Normal, NMO, NMO + CL groups consisted of three independent biological replicates from a pool of three samples. CD45 + P2ry12 + CD11b + cells were sorted, and the obtained monocytes and microglia were subjected to RNA-seq analysis.

Total RNA was isolated using Trizol reagent (15596026, Invitrogen). RNA quality was assessed by Agilent TapeStation 4000 system (Agilent Technologies, Amstelveen, The Netherlands), and RNA quantification was performed using ND-2000 Spectrophotometer (Thermo Inc., DE, USA). The construction of library was performed using QuantSeq 3ʹ mRNA-Seq Library Prep Kit (Lexogen, Inc., Austria) according to the manufacturer’s instructions. In brief, each total RNA were prepared and an oligo-dT primer containing an Illumina-compatible sequence at its 5ʹ end was hybridized to the RNA and reverse transcription was performed. After degradation of the RNA template, second strand synthesis was initiated by a random primer containing an Illumina-compatible linker sequence at its 5ʹ end. The double-stranded library was purified by using magnetic beads to remove all reaction components. The library was amplified to add the complete adapter sequences required for cluster generation. The finished library is purified from PCR components. High-throughput sequencing was performed as single-end 75 sequencing using NextSeq 550 (Illumina, Inc., USA). QuantSeq 3ʹ mRNA-Seq reads were aligned using Bowtie2^41^. Bowtie2 indices were either generated from genome assembly sequence or the representative transcript sequences for aligning to the genome and transcriptome. The alignment file was used for assembling transcripts, estimating their abundances and detecting differential expression of genes. Differentially expressed gene were determined based on counts from unique and multiple alignments using coverage in Bedtools^42^. The RC (Read Count) data were processed based on TMM + CPM normalization method using EdgeR within R (R development Core Team, 2020) using Bioconductor^43^. Gene classification was based on searches done by DAVID (http://david.abcc.ncifcrf.gov/) and Medline databases (http://www.ncbi.nlm.nih.gov/). Data mining and graphic visualization were performed using ExDEGA (Ebiogen Inc., Korea).

### Statistical analysis

Statistical significance was tested using GraphPad Prism by performing a two-tailed student-t test, ANOVA with Bonferroni’s correction, or a Kruskal–Wallis test with Bonferroni’s correction (*p < 0.05, **p < 0.01, ***p < 0.001, ****p < 0.0001; n.s., non-significant). N represents the number of animals used in the experiment.

### Supplementary Information

Supplementary Information.

## Data Availability

All high throughput sequencing data have been deposited with the Gene Expression Ombudsman (GEO) and are available under the accession number GSE235278.
