# Supplementary material for: Early involvement of peripherally derived monocytes in inflammation in an NMO-like mouse model
Source: Sci Rep. 2024 Jan 12;14:1177. doi: 10.1038/s41598-024-51759-4 (PMC10786844; doi:10.1038/s41598-024-51759-4)
Supplement: Supplementary file 1 — Supplementary Information. [file 41598_2024_51759_MOESM1_ESM.pdf]

Figure S1

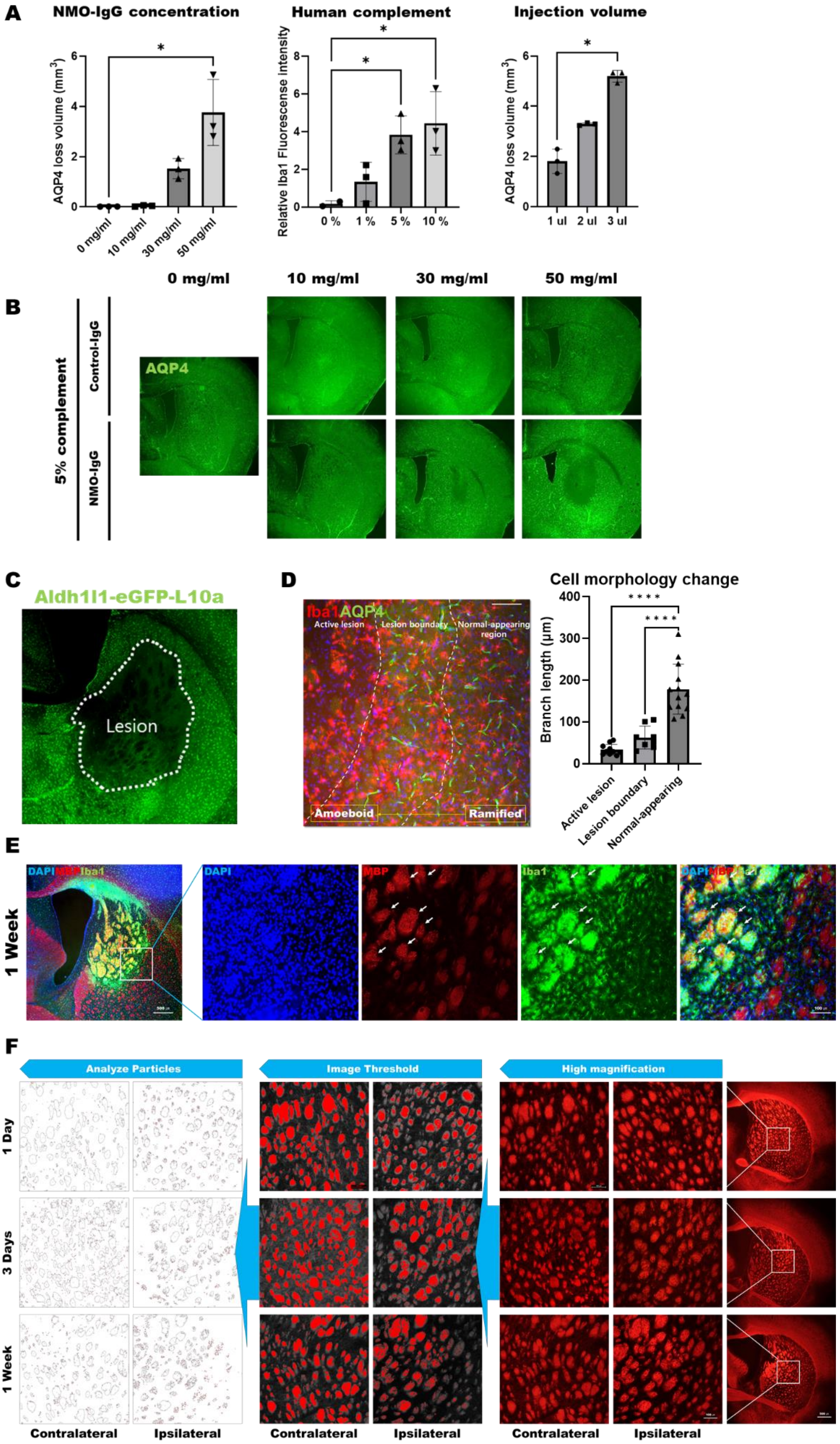
A
NMO-IgG concentration
AQP4 loss volume (mm3)
0 mg/ml
10 mg/ml
30 mg/ml
50 mg/ml
Human complement
Relative Iba1 Fluorescense intensity
0 %
1 %
5 %
10 %
Injection volume
1 ul
2 ul
3 ul
B
0 mg/ml
10 mg/ml
30 mg/ml
50 mg/ml
5% complement
Control-IgG
NMO-IgG
AQP4
C
Aldh1l1-eGFP-L10a
Lesion
D
Iba1 AQP4
Active lesion
Lesion boundary
Normal-appearing region
Amoeboid
Ramified
Cell morphology change
Branch length (μm)
Active lesion
Lesion boundary
Normal-appearing
E
1 Week
DAPI
MBP
Iba1
F
Analyze Particles
Image Threshold
High magnification
1 Day
3 Days
1 Week
Contralateral
Ipsilateral

Fig. S1. The optimization of NMO-IgG mixture. (A) Measurement of NMO-IgG concentration, inflammatory induction by human complement and determination of injection volume (Kruskal-Wallis test with Dunn's post-hoc, *p<0.05). (B) Measurement of AQP4 loss volume using various combinations of NMO-IgG and hC. (C) Induction of astrocyte depletion using a mixture of 50 mg NMO-IgG and 5% hC in Aldh1L1-eGFP-L10a TG mice. (D) Analysis of Iba1+ cell morphology based on the lesion location using Iba1+AQP4 immunostain. (E) High magnification images of the MBP and Iba1 stain. (F) Measurement process of axon bundle size.

Figure S2

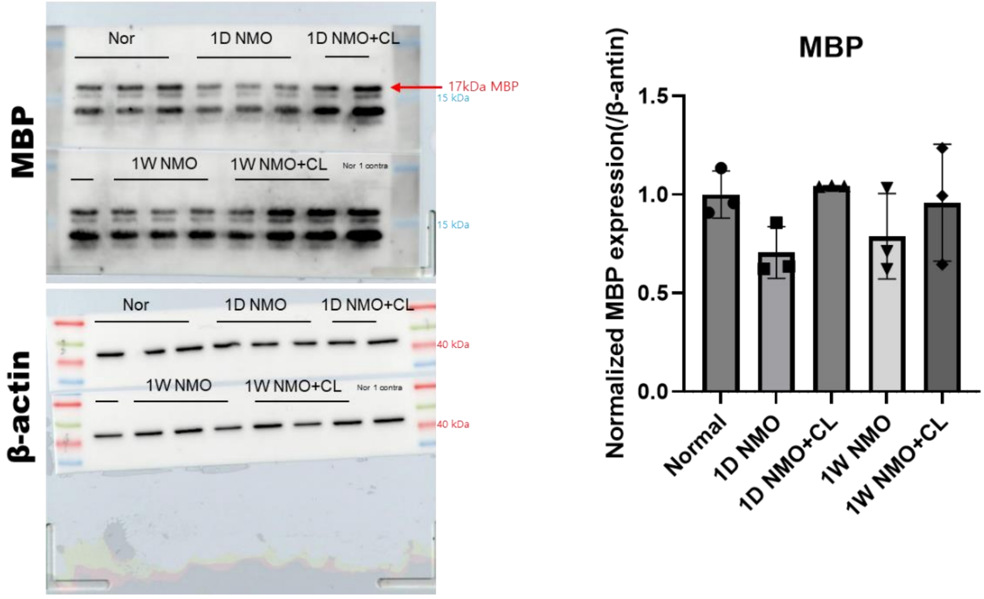

Fig. S2. After 1 day and 7 days post-injury, brain lysates from the Normal, NMO, and NMO+CL groups were subjected to SDS-PAGE to detect MBP and β-actin. The molecular sizes of 15 kDa and 40 kDa were indicated by blue and red color, while the 17 kDa MBPs were indicated by red arrows. Original blots/membranes of MBP and β-actin were obtained from different parts of the same gel.

Figure S3

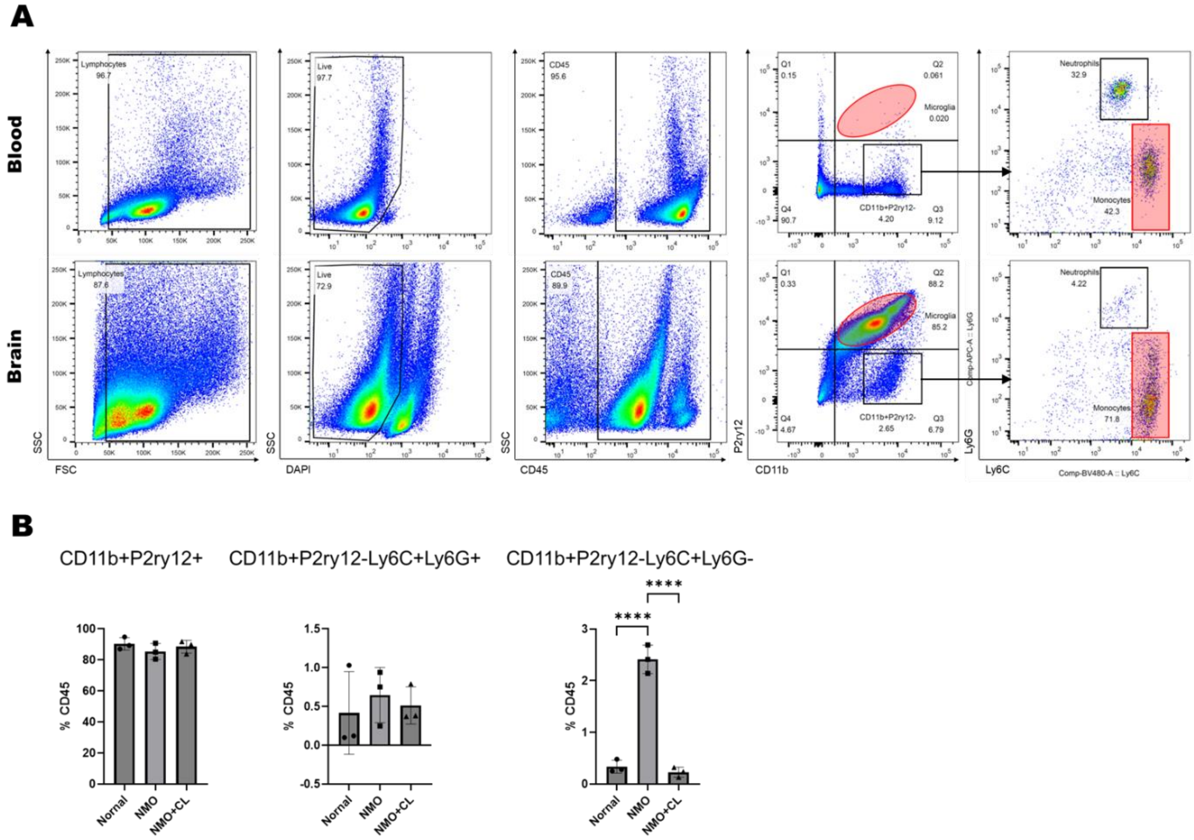

Fig. S3. Sorting of monocytes and microglia for RNA-seq analysis. (A) Immune cells were isolated from the blood and brain tissue of the Normal, NMO, and NMO+CL groups at 1 day post-injury. Monocytes and microglia were sorted based on Live/CD45+ cells, followed by gating of CD11b/P2ry12 cells and further gating of Ly6C/Ly6G cell populations from CD11b+P2ry12- cells. The transparent red squares represent sorted CD11b+P2ry12+ microglia and CD11b+P2ry12-Ly6C+Ly6G- monocytes. (B) Bar graph displaying differences in cell numbers for each cell type. (Kruskal-Wallis test with Dunn's post-hoc, ****$p<0.0001$, three independent replicates from 3-4 samples).

Figure S4

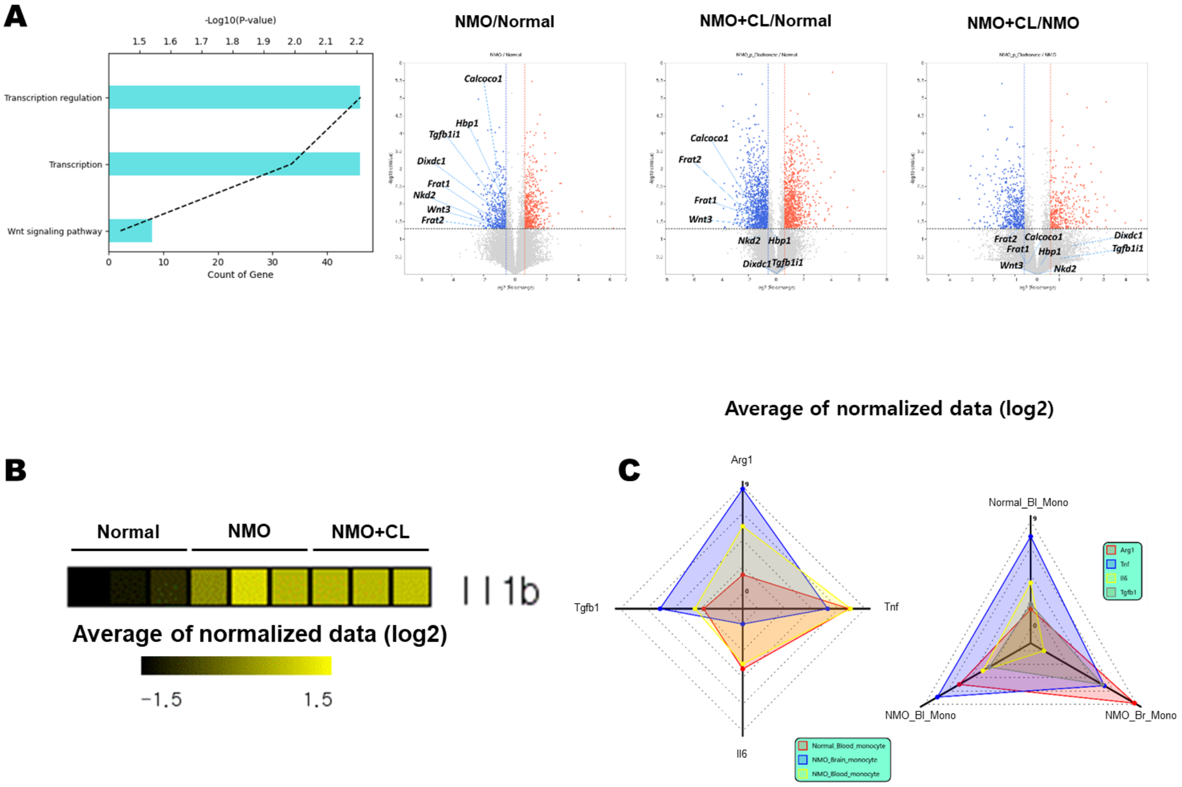

Fig. S4. Volcano plots of differential gene expression profiles showing the magnitude on the x-axis (Log2 fold change) and significance on the y-axis (-Log10 adjusted p value) for NMO vs. Normal, NMO+CL vs. Normal and NMO+CL vs. NMO. Wnt signaling-related genes are highlighted. (B) Heatmap of *Il1b* expression shows no change with clodronate treatment. (C) Pro- and Anti-inflammatory signature genes in monocyte RNA-seq.

Table 1 Groups, analysis, and sample number in animal experiments

Experiments	Analysis	Groups	Sample Number		
			1 day	3 days	1 week
Pathophysiology of NMO model	Immunofluorescence	Normal	3	3	3
		Control-IgG+hC	3	3	3
		NMO-IgG+hC	3	3	3
	PCR	Normal	3	3	3
		Control-IgG+hC	3	3	3
		NMO-IgG+hC	3	3	3
	Flow cytometry	Normal	6		
		Control-IgG+hC	6		
		NMO-IgG+hC	6		
Monocyte/marcrophages depletion	Flow cytometry	Normal	6		
		NMO	6		
		NMO+CL	5		
	PCR	Normal	3		
		NMO	3		
		NMO+CL	3		
	Immunofluorescence & LFB stain	Normal	5		5
		NMO	5		5
		NMO+CL	3		3
	Western blotting	Normal	3×3†		3×3†
		NMO	3×3†		3×3†
		NMO+CL	3×3†		3×3†
RNAseq of Monocytes	FACS	Normal	5×3†		
		NMO	5×3†		
RNAseq of Microglia	FACS	Normal	3×3†		
		NMO	3×3†		
		NMO+CL	3×3†		

†Each pooled samples × Number of independent experimental replicates
